# A case of multiple primary malignancies including peritoneal mesothelioma surviving over 11 years

**DOI:** 10.1002/ccr3.4096

**Published:** 2021-05-19

**Authors:** Ebrahim Esmati, Saeed Rezaei, Fatemeh Jafari

**Affiliations:** ^1^ Radiation Oncology Research Center (RORC) Cancer Institute Tehran University of Medical Sciences Tehran Iran

**Keywords:** lymphoma, multiple malignancies, peritoneal mesothelioma, rectal adenocarcinoma

## Abstract

Multiple primary malignancies are exceedingly rare, but they may occur sequentially in a patient, so in the follow‐up of patients with a history of malignancy, always pay attention to second and third primary malignancies as well.

## Graphical abstract

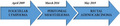

## 1 INTRODUCTION

Metachronous malignancies are exceedingly rare, but probability of second or third malignancy in a patient should be kept in mind. We present a patient with three metachronous malignancies occurring sequentially: lymphoma, mesothelioma, and rectal adenocarcinoma. Unexpectedly peritoneal mesothelioma, known as a fatal malignancy, completely regressed with chemotherapy in this patient.

Multiple primary malignancies (MPM) are defined as the occurrence of two or more malignancies in the same individual without any relationship between the tumors.^1^ Multiple primary malignancies can be divided into two categories depending on the interval between tumor diagnoses; *Synchronous* cancers are second tumors coinciding within 6 months after the first malignancy, while *metachronous* multiple malignancies are secondary cancers more than 6 months.^2^ Improving survival rates for patients with neoplastic disease due to either too early diagnosis or new therapies allows more patients to survive long enough to develop subsequent primary tumors; a literature review on 1 104 269 patients with cancer concluded that MPM prevalence is between 0.73% and 11.7%. As expected, incidence increases with aging.^3^

An individual may develop MPM in a lifetime due to genetic predisposition, environmental exposure to carcinogens, immunodeficiency, or as a serious complication of chemotherapy or radiotherapy received for first primary malignancy.

In an analysis of 161 Chinese patients with MPMs, 78 (48.4%) patients had synchronous tumors, and 83 (51.6%) patients had metachronous tumors. Most patients with MPMs were men and older (>50 years old), and adenocarcinoma was the most frequent pathology type. The most frequent location of all MPMs was the digestive system. The leading tumor association was between digestive‐digestive tumors also. Patients with synchronous tumors and MPMs of the digestive system showed a shorter survival time.^4^

**FIGURE 1:**
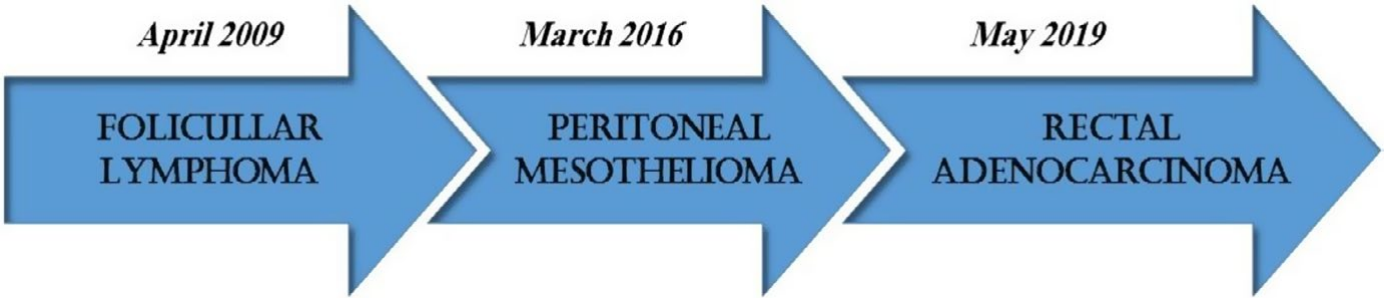
Timeline of patient

The development of multiple primary malignancies in an individual is rare and unfortunate, and such a patient's care presents specific challenges. We present a case report describing a patient with three different primary cancers over 11 years: follicular lymphoma, abdominal mesothelioma, and rectal adenocarcinoma, respectively (Figure 1). Peritoneal mesothelioma, known as a fatal malignancy, occurred without any history of asbestosis or radiation and completely regressed with systemic chemotherapy in this patient.

## 2 CASE PRESENTATION

In March 2009, a 55‐year‐old woman presented to a physician with multiple cervical nodules. The patient reported that the nodules had been present for several weeks. The patient's medical history also included hyperlipidemia and hypertension. She was a housewife and had no discrete occupational exposure to chemicals, etc Family history included no known instance of cancer. During the physical examination, multiple bilateral lymphadenopathies of the anterior cervical chain and supraclavicular were detected on palpation, which was not tender and was neither erythematous nor warm to the touch. Findings of the remainder of the physical examination were normal. The patient's medical history revealed no fever, weight loss, and night sweats. A cervical excisional biopsy was done, and the pathologist reported "*Follicular Lymphoma grade ШB."*

Further evaluations, including chest/abdomen CT and cervical sonography and bone marrow aspiration/biopsy, detected no more findings (stage I follicular lymphoma). The patient received standard treatment, including four R‐CHOP regimen courses (cyclophosphamide, doxorubicin, vincristine, and prednisone plus the monoclonal antibody rituximab) followed by involved filed radiation therapy (IFRT). Cervical lymph nodes were completely resolved in neck CT after completion of chemotherapy and radiotherapy. Routine, annual visits, and physical examination were recommended and continued up to April 2016.

**FIGURE 2:**
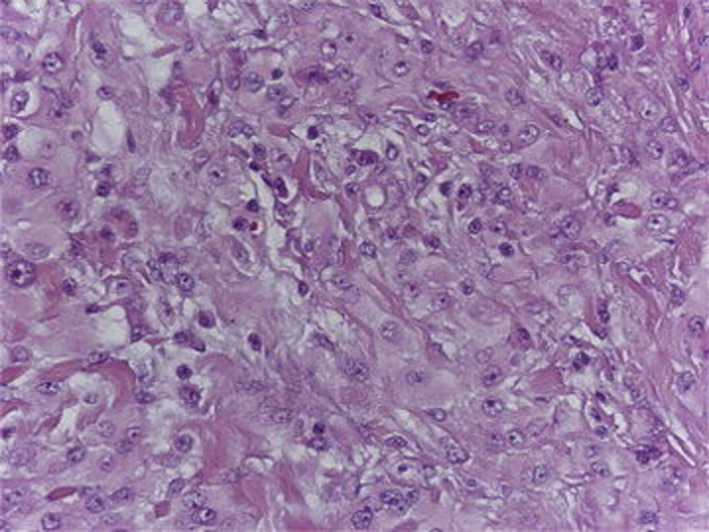
Peritoneal mesothelioma

In April 2016, an approximately 4 centimeter mass of the right inguinal region was detected in the physical examination, which was not tender and was neither erythematous nor warm on palpation. Abdominopelvic CT showed multiple lymphadenopathies of para‐aorta and iliac chains with a maximum SAD of 14 millimeters in favor of metastasis. Chest CT had no abnormal findings. Excisional biopsy of inguinal/lower abdomen mass was done, and pathology/IHC study made the diagnosis of "*Mesothelioma"* (positive for Pan‐CK, CK7, calretinin, and WT‐1 and negative for CD20, P63, TTF1, and CEA), which was confirmed by two pathologists (Figure 2).

The patient received a cisplatin+pemetrexed chemotherapy regimen. Abdominopelvic CT after completion of chemotherapy showed that inguinal mass and lymphadenopathies are completely resolved. Routine, periodic visits, and physical examination were recommended and continued up to May 2019.

In May 2019, an incidental finding of pelvic CT reported a mass‐like lesion of rectum and fat stranding of around. A colonoscopic study revealed a large ulcerative tumor of the rectum. Complementary studies confirmed "*Adenocarcinoma of Lower Rectum T3 N2 M0."* The patient received the standard treatment of rectal adenocarcinoma (neoadjuvant chemoradiation followed by surgery).

Our patient is a 66‐year‐old woman and is alive 11 years after the diagnosis of follicular lymphoma, 4 years after diagnosis of mesothelioma, and 1 year after diagnosis of rectal adenocarcinoma.

## 3 DISCUSSION

Metachronous multiple primary neoplasms are uncommon clinical entities that often require intensive medical care. The prevalence of this phenomenon has been increasing in recent years because of the increased survival rate associated with cancers previously considered fatal (ie, allowing the patient time to develop a second or third malignancy).

It is difficult to accurately determine the prevalence of multiple primary neoplasms because only available data come from studies based on case reports. Koutsopoulos et al tabulated the results of an extensive literature search for multiple primary cancers and found 42 cases of 3 or more cancers reported between 1949 and 2003, proving that the entity is rare. Important epidemiologic parameters, such as incidence, prevalence, and prognosis, would almost certainly depend on the particular combination of cancers.^5^ A retrospective analysis of 72 Chinese patients with MPMs showed that those presenting with metachronous cancers have higher incidence and better prognosis.^6^

Malignant mesothelioma is a rare malignancy mainly caused by occupational or environmental asbestos exposure and arises mostly from pleura, although it rarely originates from other mesothelial structures such as the peritoneum and pericardium. Other possible risk factors are radiation exposure and genetic predisposition.^7^ Interestingly, 50% of patients with peritoneal malignant mesothelioma have no documented asbestos exposure. Although overall mesothelioma is more common in men, higher proportions of women develop peritoneal mesothelioma.^8^, ^9^

The frequency of non‐Hodgkin lymphoma (including follicular lymphoma) is greatly increased in immunocompromised patients; the two most common clinical circumstances are HIV‐infected patients and solid organ transplant recipients; both are associated with prolonged immunosuppression.^10^, ^11^ Many occupations have been associated with a higher risk of developing non‐Hodgkin lymphoma (NHL). A pooled analysis from the Inter‐Lymph Consortium demonstrated an increased incidence of NHL in farmworkers, women's hairdressers, charworkers/cleaners, spray painters, electrical wiremen, and carpenters. Exposure to solvents such as benzene, toluene, xylene, and styrene is linked to follicular lymphoma development (FL).^12^

Increasing age, male sex, and excessive alcohol use have been associated with an increased risk of rectal cancer.^13^

The risk factors mentioned above are all related to each of the cancers individually, but it is not clear whether any of these environmental, genetic, and immunosuppressive factors are involved in MPMs or not.

Wei Xie reported a case of a 61‐year‐old male presenting synchronous primary colonic adenocarcinoma and malignant mesothelioma without any documented history of the source of asbestos exposure.^14^ However, to the best of our knowledge, the present report is the first to discuss a rare case of 3 primary cancers that includes equally rare mesothelioma of the peritoneum without any known predisposing factor (eg, radiation or asbestos exposure). The particular combination of cancers should be examined in an attempt to explain the occurrence through the elucidation of a common risk factor or perhaps genetic predisposition; regarding the relationship between asbestos exposure and tumors of the hematopoietic system, we should know that development of NHL and asbestos‐related mesothelioma in the same patient has repeatedly been observed.^15^, ^16^, ^17^ A small study has also supported an association between occupational exposure to asbestos and colon cancer incidence in men.^18^ Our patient had neither prior history of asbestos exposure (as a probable common risk factor of mesothelioma, lymphoma, and rectal adenocarcinoma) nor abdominal radiation, but the genetic study was not accomplished. A study conducted in 2018 by Hung et al on 88 patients with peritoneal mesothelioma showed that 13% of patients are anaplastic lymphoma kinase (ALK) positive by immunohistochemistry and in three out of eleven patients; ALK rearrangement was detected by fluorescence in situ hybridization (FISH) as well.^19^ Besides, some genetic studies of colorectal cancer have shown that ALK, ROS1, and NTRK fusions occur in 0.2% to 2.4% of patients.^20^ Although the genetic study was not available for our patient, hypothetically, ALK rearrangement or other unknown genetic disorders may be a common finding of peritoneal mesothelioma and rectal adenocarcinoma in our patient.

The lessons we learn from this patient are as follows:

1. In the follow‐up of patients with a history of malignancy, always pay attention to second and third primary malignancies.
2. Although mesothelioma occurs mainly in the pleura and contact with asbestos or radiation, it can rarely occur in the peritoneum without a history of contact with asbestos or radiation. Peritoneal mesothelioma is highly lethal and has a poor prognosis, but sometimes the patient recovers with systemic chemotherapy such as pemetrexed/cisplatin and can live for several years after that.
3. In patients with multiple primary malignancies (synchronous or metachronous) and malignancy in the absence of a well‐known risk factor (eg, mesothelioma in the absence of asbestos or radiation exposure), a genetic study may help to understand the cause better.

In conclusion, a review of the literature showed that multiple primary malignancies are exceedingly rare. However, they may occur sequentially in a patient same as ours (ie, non‐Hodgkin lymphoma then peritoneal mesothelioma and finally rectal adenocarcinoma). Peritoneal mesothelioma, known as a fatal malignancy, may completely regress with chemotherapy and cause survival for many years.

## CONFLICT OF INTEREST

None declared.

## AUTHOR CONTRIBUTIONS

EE: involved in revising the manuscript for important intellectual content. SR: made contributions to design the manuscript and analysis and interpretation of data. FJ: involved in final revision of the manuscript.

## ETHICAL APPROVAL

We safeguard privacy rights and confidential information, balancing an individual's right to privacy with the needs of our institutions to collect, analyze, record, maintain, use, and disseminate information.

## Data Availability

The data that support the findings of this study are available from the corresponding author.
